# Vitamin D Nanoliposomes to Improve Solubility, Stability, and Uptake Across Intestinal Barrier

**DOI:** 10.3390/pharmaceutics17101244

**Published:** 2025-09-23

**Authors:** Cosimo Landi, Elisa Landucci, Costanza Mazzantini, Rebecca Castellacci, Maria Camilla Bergonzi

**Affiliations:** 1 Department of Chemistry, University of Florence, Via Ugo Schiff 6, Sesto Fiorentino, 50019 Florence, Italy; cosimo.landi@unifi.it (C.L.); rebecca.castellacci@unifi.it (R.C.); 2 Department of Health Sciences, University of Florence, Viale Pieraccini 6, 50139 Firenze, Italy; elisa.landucci@unifi.it (E.L.); costanza.mazzantini@unifi.it (C.M.)

**Keywords:** Vitamin D, nanoliposomes, TPGS, drug delivery, dissolution, Caco-2 uptake, cytotoxicity

## Abstract

**Background/Objectives**: Vitamin D (VD) is a fat-soluble vitamin essential for bone health, and calcium and phosphorus absorption. Recently, new interesting functions are reported such as neuroprotective activity, regulatory roles in the immune system, and protective effects in cancer patients. However, the lipophilic nature of VD represents a limitation, as it is associated with low solubility and poor absorption; additionally, VD exhibits poor stability. **Methods**: Two nanoliposomes containing VD, conventional (LP-VD) and conjugated with D-α-tocopheryl polyethylene glycol 1000 succinate (TPGS, LPT-VD), were developed. The physical and chemical stability during the storage and gastrointestinal stability, the dissolution profile, the cytotoxicity and the Caco-2 cellular uptake were investigated. Nanoliposomes were fully characterized determining sizes, PdI, Zeta potential, encapsulation efficiency and recovery and they were lyophilized to improve stability. Subsequently, the freeze-dried liposomes were encapsulated in hard gelatin capsules to mimic an oral dosage form, and they were subjected to dissolution test. **Results**: LP-VD exhibited an average size of 85.50 ± 5.70 nm, a PdI of 0.24 ± 0.06, and a ZP of −20.90 ± 4.37 mV. LPT-VD showed an average size of 61.70 ± 3.90 nm, a PdI of 0.26 ± 0.02, and a ZP of −9.45 ± 2.99 mV. The EE% values were 95.76 ± 1.26% and 97.54 ± 3.24% for LP-VD and LPT-VD, respectively. Both nanoliposomes solubilized 2 mg/mL of VD and improved both its storage stability and stability in aqueous and gastrointestinal environment. The freeze-dried products guarantee constant chemical-physical parameters for 28 days at 25 °C. VD dissolution profile was improved. **Conclusions**: Nanoliposomes, in particular LPT-VD, showed the best results in terms of chemical stability, dissolution profile, and Caco-2 cellular uptake, confirming the stabilization, bioenhancer properties and P-gp inhibition capabilities of TPGS.

## 1. Introduction

Vitamin D (VD) is an endogenous molecule synthesized by the body upon exposure to UV radiation. It can also be obtained through dietary intake though it is found in only a limited number of foods such as oily fish (salmon, sardines, trout) red meat, egg yolks, fortified foods, such as some fat spreads and breakfast cereals [1]. Despite these sources, low blood levels of VD are frequently observed, particularly in populations living at higher latitudes [2]. VD deficiency has long been recognized as a major public health issue. As early as the 17th century, it was linked to diseases such as rickets and osteomalacia [3]. More recent studies have highlighted vitamin D exerts pleiotropic actions, including neuroprotective effects in neurodegenerative diseases such as Alzheimer’s disease, Parkinson’s disease, and multiple sclerosis [4,5], involvement in calcium and phosphorus homeostasis [2], immune system regulation [6], and a protective role in cancer patients [7,8,9]. Given its importance, addressing VD deficiency is crucial. However, due to its lipophilic nature, it exhibits low water solubility, resulting in poor absorption [10,11]. Its bioavailability largely depends on dietary fats and bile salts, which help emulsify the vitamin in gastrointestinal fluid [2]. Commercial products often combine VD with natural oils to simulate this effect and improve absorption compared to the powder form.

In recent decades, research in pharmaceutical technology has increasingly focused on the development of innovative drug delivery systems (DDS). DDS are designed to transport bioactive molecules overcoming the limitations of conventional dosage forms, such as poor pharmacokinetics (absorption, distribution, metabolism, and excretion), low patient compliance, poor stability, and significant side effects [12,13,14,15,16,17,18]. Thanks to DDS, it is now possible to reduce absorption variability and increase the amount of VD absorbed [19]. These properties combined with their biocompatibility make liposomes particularly suitable as carriers for natural compounds with poor in vivo bioavailability, low chemical stability and limited solubility in aqueous media [15,17,20,21,22]. Bi et al. developed liposomes for the topical administration of VD as an anti-aging agent, enhancing its transdermal absorption and stability [23]. Similarly, Mohammadi et al. [24] demonstrated that nanoliposomes can protect VD from oxidation in aqueous food products and proposed their use in beverage fortification.

In this study, nanoliposomes were selected as delivery system to improve VD aqueous solubility, stability, dissolution, and intestinal absorption. Two formulations were developed: one composed of cholesterol and phosphatidylcholine (LP-VD), and another containing also D-α-tocopheryl polyethylene glycol 1000 succinate (TPGS) (LPT-VD), a polymer known for its antioxidant properties and its ability to inhibit the P-glycoprotein (P-gp) efflux pump, which plays a key role in multidrug resistance in cancer cells. Furthermore, several studies have reported that TPGS liposomes can increase drug absorption after oral administration [15,25].

Both liposomal formulations were prepared using the lipid film hydration method and characterized via Light Scattering techniques. The recovery percentage and encapsulation efficiency were evaluated. A storage stability study on LP-VD and LPT-VD at 4 °C was conducted, and the freeze-drying process was evaluated to further improve the chemical stability of the formulations. Gastrointestinal stability was also considered. The lyophilized liposomes were encapsulated into hard gelatin capsules as oral dosage form and the impact of the formulations on the release profile of VD was assessed with the dissolution test. Furthermore, the safety and uptake of VD liposomes across human colon adenocarcinoma (Caco-2) monolayer cells were evaluated [26].

## 2. Materials and Methods

### 2.1. Chemicals and Reagents

Vitamin D (Cholecalciferol, purity ≥98%, VD, no. C9756), Cholesterol (CH, purity ≥92.5%, no. C8503), Coumarin 6 (6C, purity 98%, no. 442631), Phosphate-buffered saline pH 7.4 (PBS, no. P3813), D-α-Tocopheryl polyethylene glycol 1000 succinate (TPGS, no. 57668), Sodium dodecyl sulfate (SDS, no. 75746), purity ≥99.0%, Tween 80 (no. 8.17061), Acetonitrile HPLC grade (no. 34851), Dichloromethane (CH2Cl2, no. 02575), Dimethyl sulfoxide (DMSO, purity ≥98%, no. 472301), Ethanol 96% (no. 24105), Methanol HPLC grade (no. 34860) were purchased from Sigma-Aldrich (Milan, Italy). Phosphatidylcoline 90 G (PC, no. 97281-47-5), purity 94–102%, was purchased from Phospholipid GmbH (Cologne, Germany). Ultrapure water was produced using a Merck Millipore’s Sim-plicity^®®^ UV Water Purification System (Merck KGaA, Darmstadt, Germany).

### 2.2. Analytical Method

The quali-quantitative determination of VD was carried out using a High-Performance Liquid Chromatograph (HPLC), HP 1200, equipped with an autosampler and a Diode Array Detector (DAD), (Agilent Technology, Santa Clara, CA, USA) operating in the UV-visible region. The chromatographic separation was achieved using a Kinetex C18 column (150 × 4.6 mm, 5 µm Phenomenex, Bologna, Italy). The analytical method involves a 20-min gradient elution, with a flow rate of 0.6 mL/min. The mobile phase is composed of two eluents: Millipore water acidified with formic acid (0.007% p/v pH 3.2), and HPLC grade acetonitrile, CH_3_CN. The following multistep linear gradient was used: solvent B increased from 80% to 100% in 5 min, then remained at 100% for 13 min then returned to 80% in 2 min. Equilibration time 10 min. The chromatographic profile was recorded at a wavelength of 264 nm (Supplementary Materials, Figure S1). Under these conditions, VD exhibits a retention time of approximately 10 min.

6C quali-quantitative determination was performed using the same instruments and the same method described before for VD, but the chromatographic profile was recorded at 444 nm.

Diluting stock solutions in CH_3_OH (0.524 mg/mL for VD and 0.102 mg/mL for 6C), standard solutions were freshly prepared. To quantify each compound, an external standard method was applied using a regression curve and analyses were performed in triplicate. Results were expressed as the mean ± SD of the 3 experiments.

All the compounds showed a linear response: VD from 0.016 µg to 3.931 µg; 6C from 0.025 μg to 0.408 μg. All the curves had coefficients of linear correlation R^2^ ≥ 0.999.

### 2.3. Preparation of Liposomes (LP), TPGS-Coated Liposomes (LPT), VD-Loaded Liposomes (LP-VD) and VD-Loaded TPGS-Coated Liposomes (LPT-VD)

Empty nanoliposomes (LP) and TPGS-coated nanoliposomes (LPT) were prepared with the thin-layer evaporation method. The constituents of LP were PC and CH in a weight ratio of 3:1. This ratio was selected after testing two different ratios (3:1 and 5:1), as it resulted in smaller particle size and lower PdI. LPT contained PC, CH, and TPGS. Different weight ratios were tested for LPT such as 3:1:1, 3:1:3 and 3:3:1 and the ratio 3:1:3 was chosen. The lipid phase was dissolved in 5 mL of CH_2_Cl_2_. Then, the organic solvents were evaporated, and the lipid film was hydrated with 10 mL of deionized water. The system was maintained in a water bath at 50 °C under magnetic stirring for 30 min. The same method was applied to LP-VD and LPT-VD preparations, with the addition of VD to the organic phase. VD final concentration resulted in 2 mg/mL. To reduce particle size and achieve a more homogeneous sample, an ultrasonic sonicator was used, performing an initial 5-min session followed by a 2-min session. Sonication was conducted with 2-s intervals at an intensity of 50% for LP and LP-VD, and 40% for LPT and LPT-VD. During the sonication process, the sample was maintained in an ice bath to prevent overheating and lipid degradation. The composition of all nanoliposomes was reported in Table 1.

### 2.4. Preparation of 6C-Loaded Liposomes (LP-6C) and 6C-Loaded TPGS-Coated Liposomes (LPT-6C)

6-coumarin (6C) loaded liposomes (LP-6C and LPT-6C) were prepared using the same methods used for VD liposomes. In both formulations 5 mg of 6C were dissolved in the lipid phase, reaching final concentration of 0.5 mg/mL.

### 2.5. Lyophilization Method

Both VD-loaded nanoliposomes were freeze-dried. Different aliquots of the preparation (1, 2, and 5 mL) were put into glass vials, which were then sealed, wrapped with parafilm, and frozen under dark conditions. Once the freezing phase was completed, the samples were placed in the lyophilizer (Lyovac GT 2, Leybold Heraeus, Köln, Germany) for 24 h with a pressure of process of −1.0 bar. After reconstitution of the product with original volume of water, changes in terms of mean diameter, ζ-potential and VD recovery percentage, were evaluated.

### 2.6. Physical Characterization

Physical characterization (average diameter and polydispersity index, PdI) was carried out by Dynamic Light Scattering technique (DLS), employing the Z-sizer Nanoseries ZS90 (Malvern Molecules Instrument, Worcestershire, UK), after a 4-fold dilution with dezionized water. The Zeta potential (ZP) was determined by Electrophoretic Light Scattering technique (ELS), using the same instrument. The results were expressed as an average of three measurements.

### 2.7. Encapsulation Efficiency (EE%) and Recovery% (R%)

The EE% of LP-VD and LPT-VD was determined using a dialysis bag (Cut-off 3.5–5 kDa) adequately hydrated in distilled water before use. Two mL of the formulation were inserted into the bag and kept under magnetic stirring for 1 h, at room temperature, inside 1 L of distilled water. Subsequently, the dispersion was taken from the bag and diluted 10 times with HPLC-grade MeOH; then the sample was kept in the ultrasonic bath for 10 min and ultracentrifuged for 10 min at a speed of 14,000 rpm. The supernatant was analyzed by HPLC-DAD. The evaluation of the EE% was carried out using the following equation:

(1) $$EE\%=\frac{mg\;encapsulated\;VD}{mg\;total\;VD}\;\times 100.$$

To calculate the R%, the formulations were diluted 10 times with MeOH, kept in the ultrasonic bath for 10 min, ultracentrifuged for 10 min at 14,000 rpm and, subsequently, analyzed by HPLC-DAD. The evaluation of the R% was carried out using the following equation:

(2) $$Recovery\%=\frac{total\;recovered\;VD\;(mg)}{weighted\;VD\;(mg)}\;\times 100.$$

### 2.8. Stability Studies

#### 2.8.1. Storage Stability Studies

The chemical stability study was carried out on a water (1% *v*/*v* CH_3_OH) VD solution, as well as on VD liposomal formulations (LP-VD, LPT-VD) stored at +4 °C as aqueous dispersions, and as lyophilized products stored at room temperature (+25 °C) under dark conditions. The VD solution was prepared by dissolving the compound in CH_3_OH (2 mg/mL), and subsequently diluted 100-fold with water. Between analyses, the sample was stored in a refrigerator at +4 °C. All samples were protected from the light; the analyses were performed in triplicate and the R% was determined as described before.

To investigate the physical stability of LP-VD and LPT-VD, the colloidal dispersions were transferred into glass bottles sealed with plastic caps. Both were stored as a suspension at 4 °C for one month under dark conditions for 28 days. Before the analysis, the liposomes were diluted with deionized water (FD5).

The lyophilized products were stored at room temperature (+25 °C) under dark conditions for 20 days. Before the analysis they were rehydrated and analyzed. The average diameter, PdI, Zeta potential and any visual phenomena of instability were evaluated. Each parameter was measured in triplicate.

#### 2.8.2. Gastrointestinal Stability

Furthermore, the physical stability of liposomes was performed in simulated gastrointestinal environment. LP-VD and LPT-VD were incubated at 37 °C in simulated gastric fluid (SGF) followed by simulated intestinal fluid (SIF). SGF consisted of 2 g of NaCl diluted into 1 L of purified water. The pH was adjusted to 1.2 with about 7 mL of HCl 1 N. LP-VD and LPT-VD were mixed with SGF (final ratio 1:1 *v*/*v*) and maintained at 37 °C under continuous shaking (100 rpm) for 2 h. Then, the digested samples were incubated for 4 h at 37 °C with SIF (final ratio 1:1 *v*/*v*). SIF consisted of 6.8 g of KH_2_PO_4_ and 0.9 g of NaOH diluted into 100 mL of purified water. During the incubation in SGF and in SIF the samples were collected, diluted with distilled water at a dilution factor (FD) of 5 and analyzed by DLS to assess particle size and PdI. The studies were performed in triplicate [19].

### 2.9. Dissolution Test

Freeze-dried LP-VD and LPT-VD and VD were subjected to the dissolution test to simulate their behavior in the gastrointestinal environment. “0” type capsules were filled with VD (20 mg), instead, the two freeze-dried samples were placed in “00 type” capsules, each one containing a quantity of formulation corresponding to 20 mg of VD, and put in a basket connected to an AT7 semi-automated dissolution tester (SOTAX AG, Aesch, Switzerland). Each basket was inserted in a vessel containing water (900 mL) with 0.3% w/v of sodium dodecyl sulfate (SDS) at 37 °C and kept on rotation for 48 h. During the test 1 mL aliquots were taken, replacing them with water with 0.3% of SDS, and analyzed by HPLC.

### 2.10. In Vitro Cellular Study

#### 2.10.1. Cell Culture Conditions

The human Caco-2 cell line was purchased from ATCC (HTB-37; Lotto 70057475; Cell Lines Service) and maintained in culture using high-glucose DMEM medium (D5671; Sigma-Merck, Milan, Italy) supplemented with 10% fetal bovine serum (F7524; Sigma-Merck, Milan, Italy), 1% glutamine, and 1% penicillin/streptomycin (P/S). Cells were incubated at 37 °C in a 5% CO_2_ atmosphere. Cells were plated in Petri dishes (p100), and the culture medium was changed twice a week. Once they reached approximately 70–80% confluence, the cells were split at a 1:3 ratio into new Petri dishes (p100).

#### 2.10.2. Uptake Study

To assess intracellular 6C content, Caco-2 cells were exposed for 2 h to the LP-6C and LPT-6C loaded with 0.5 mg/mL of 6C and diluted 1: 1000 into a medium, and to a saturated solution of fluorescent probe. At the end of the treatment, cells were fixed in 4% formaldehyde (pH 7.4) for 15 min. Cellular uptake was investigated by fluorescence microscopy using liposomes labeled with 6C, with a high-pressure mercury vapor lamp, 20× objective, NA = 0.75 (OLYMPUS BX3-CBH/U-MCZ, Evident Europe GmbH, Milan, Italy). Filter set: excitation 365 nm emission 400 nm high pass DAPI, excitation 485 nm emission 524 nm.

Before the uptake test, to evaluate the stability of probe inside the nanoliposomes the in vitro release test was conducted using a dialysis bag (cellulose membranes, cut-off 3.5–5 kDa, SpectrumLabs, San Francisco, CA, USA). The dialysis bag was filled with 2 mL of undiluted formulation, sealed at both ends with appropriate clips, and placed in a beaker containing 200 mL of the release medium, maintained at 37 °C under magnetic stirring at 100 rpm. Phosphate-buffered saline (PBS, pH 7.4) containing 1% of Tween 80 was used as acceptor medium. Samples of 1 mL were collected at intervals of 1, 2, 4, 6 h and replaced with an equal volume of the PBS/Tween 80 mixture to maintain initial sink conditions. The samples collected were analyzed using HPLC-DAD to determine the amount of VD released over time.

#### 2.10.3. Cytotoxicity

3-(4,5-dimethylthiazol-2-yl)-2,5-diphenyltetrazolium bromide (MTT) Assay.

To assess cell viability after VD, LP-VD, LPT-VD, 6C, LP-6C and LPT-6C exposure, the MTT assay was performed in Caco-2 cell line. Cells were seeded in a 24-well plate and grown at 37 °C in an atmosphere of 5% CO_2_ in DMEM medium. When the cells were approximately 70–80% confluent they were exposed for 2 h to VD free at the concentrations of 0.02 and 0.01 mg/mL and to LP-VD and LPT-VD (both loaded with VD at a concentration of 2 mg/mL) diluted 1:100 and 1:200 into a culture medium consisting of HBSS buffer supplemented with Ca^2+^, Mg^2+^, and 25 mM Hepes at pH 7.4. Similarly, Caco-2 were exposed for 2 h to 6C free at the concentration of 5 μg/mL and 2.5 μg/mL and to LP-6C and LPT-6C, both loaded with 0.5 mg/mL of 6C and diluted 1:100 and 1:200 into HBSS plus Hepes medium. The medium of each well was separated from the cells and stored for lactate dehydrogenase (LDH) assay, and cells were treated with 1 mg/mL of MTT for 15 min at 37 °C and 5% CO_2_. Finally, DMSO was added to dissolve MTT formation and absorbance was measured at 550 and 690 nm. Cell viability was expressed as a percentage compared to the cells incubated only with HBSS medium (CRL). Triton X-100 was employed in the MTT assay as the negative control since its detergent action disrupts the cells.

### 2.11. LDH Assay

Cytotoxicity after VD, LP-VD, LPT-VD, 6C, LP-6C and LPT-6C exposure was verified with LDH assay. Damage in Caco-2 cells was quantitatively assessed by measuring the amount of LDH released by the damaged cells into the culture medium, 2 h after VD, 6C and liposomes exposure, by adding catalyst and dye solutions of a Cytotoxicity Detection Kit (LDH) (Roche Diagnostics, Indianapolis, IN, USA). The absorbance values were recorded at 490 nm and 690 nm. Cytotoxicity was expressed as a percentage compared to the maximum LDH release in the presence of Triton X-100 (TX).

### 2.12. Statistical Analysis

Results of Caco-2 cell viability are expressed as mean ± SEM. The statistical significance of differences between control and treatment groups in MTT and LDH assays was analyzed using one-way ANOVA followed by a post hoc Dunnett’s test for multiple comparisons. All statistical analyses were performed using the GraphPad Prism v. 8 for Windows (GraphPad Software, San Diego, CA, USA) and a probability value (p) <0.05 was considered significant.

## 3. Results and Discussion

### 3.1. Preparation and Characterization of Liposomes

The bilayer of nanoliposomes contains CH and PC, two of the most common lipids used for liposome preparations. CH can modify the fluidity, thickness, rigidity, and stability of the bilayer, as well as influence the encapsulation efficiency. CH increases membrane rigidity by enhancing the organization of phospholipids within the bilayer. However, excessive amounts can reduce the release of the encapsulated substance [27,28,29]. TPGS is a water-soluble nonionic surfactant synthesized by esterification of vitamin E succinate with the PEG chain. It increases emulsification process, solubilization and bioavailability of lipophilic substances. It is recognized as safe, and it can be administrated via oral, topic, nasal and parenteral route. Several studies demonstrated that the presence of TPGS in the membrane induces an increase in cellular uptake, enhances transcellular passage, improves oral bioavailability, and extends the drug’s half-life. Compared to PEG, TPGS offers numerous advantages, one of the most significant being its ability to inhibit the activity of P-gp, which acts as an efflux pump and constitutes a key mechanism of resistance to anticancer drugs. Additionally, a unique property of TPGS is its antioxidant activity, which can play a protective role against oxidative degradation of numerous drugs and lipids caused by reactive oxygen species (ROS) [14,24]. For the optimization of LP, two different PC:CH gravimetric ratios were tested (5:1 and 3:1). Both formulations exhibited similar sizes and PdI (61.14 ± 1.29 and 68.98 ± 1.90 nm and 0.24 ± 0.01 and 0.23 ± 0.01 as PdI, respectively); therefore, the study was conducted with the formulation containing a lower lipid content. For LPT optimization different gravimetric ratios between PC:CH:TPGS were considered (3:1:1, 3:1:3, 3:3:1 Table 2).

Based on physical parameters and stability, the formulation with 3:1:3 gravimetric ratio was selected for VD delivery (Table 3). Both liposomes are loaded with 2 mg/mL of VD, improving the solubility of VD in water by 100 times. The physical parameters of the formulations are reported in Table 3 and in Supplementary Materials, Figures S2–S5. No significant changes in size and size distribution were evidenced; empty nanoliposomes have similar dimensions between 60 and 70 nm and low PdI. VD loading causes a moderate increase in size. Furthermore, no significant change occurred on ZP with the incorporation of VD, ensuring minimal aggregation and monodispersity of nanoliposomes in biological media, consistent with the low PdI values.

### 3.2. Encapsulation Efficiency (EE%)

Both formulations exhibited high encapsulation efficiencies, 95.76 ± 1.26% for LP-VD and 97.54 ± 3.24% for LPT-VD. This is likely due to the lipophilic nature of VD, which has a strong affinity for the lipid bilayers. High encapsulation efficiency makes lipophilic compounds more stable against hydrolytic degradation, oxidation and it allows for a slow release, as previously confirmed for other lipophilic vitamins [24,30].

### 3.3. Storage Stability Studies

#### 3.3.1. Chemical Stability

The stability in aqueous media of VD is reduced in relation to various factors, including pH, temperature, medium, concentration, light, oxygen, and the presence of other substances [31]. The chemical stability study was conducted on LP-VD and LPT-VD, stored both as a colloidal dispersion at +4 °C and as freeze-dried product at +25 °C and the results were compared with a VD aqueous solution. VD remains stable in solution for 4 h, but after one day a noticeable degradation (24%) was observed. The recovery reached 39% after 4 days (Table 4). These data proved the VD aqueous instability, even when stored at +4 °C and in a light-protected environment [31]. Abbasi reported that VD rapidly degrades in the traditional media (water/ethanol) [32].

The study was extended over 28 days for LP-VD and LPT-VD, demonstrating significant improvements in the compound’s stability inside the formulations (Table 4). In particular, after 2 weeks R% remained close to 100% in LPT-VD, while it slightly decreases in LP-VD until 93% after 4 weeks. Both liposomal formulations protect VD from degradation phenomena in solution at 4 °C, as also reported by Mohammadi [24], and the presence of TPGS provides the best performance due to the presence of PEG moiety [15,33].

The freeze-drying is a common technique used to increase the stability of liposomes. Lyophilization was also considered hypothesizing the preparation of a solid commercial product for oral use. Furthermore, the solid product is easier to formulate and store than a colloidal dispersion.

Liposomes were freeze-dried and stored at +25 °C for 28 days. Before analysis to evaluate their chemical stability, the freeze-dried liposomes were re-dispersed in purified water to evaluate R%.

**Figure 1:**
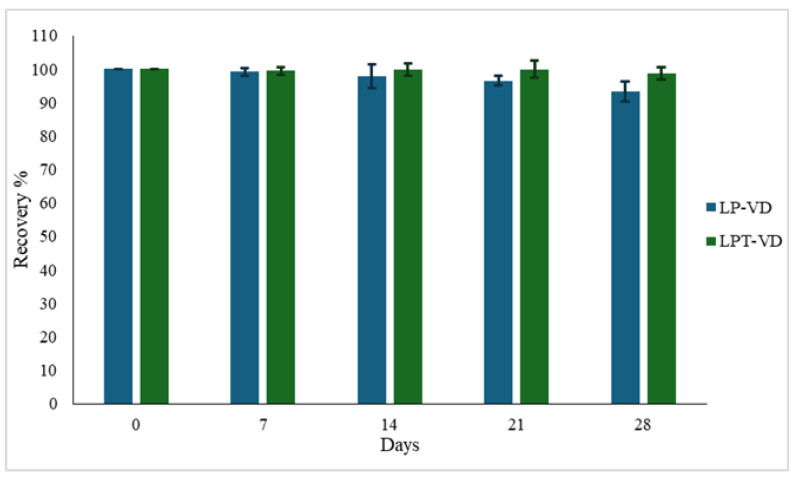
VD recovery percentage (R%) in lyophilized LP-VD and LPT-VD, stored at +25 °C. Data are reported as mean ± SD of n = 3.

In both formulations, R% is comparable to those observed in nanoliposomes stored as dispersion, with the advantage that in this case the freeze-dried is a solid product and it is stored at room temperature (Figure 1). The lyophilization process effectively preserved VD chemical stability within the liposomal structures and the lyophilized liposomes, particularly those with TPGS, are a key formulation for enhance the stability of VD.

#### 3.3.2. Physical Stability

The physical stability was investigated monitoring over time the changes in physical parameters. The study was carried out on LP-VD and LPT-VD stored as colloidal dispersion at +4 °C for 28 days. The dimensions ranged from 84 nm to 98 nm for LP-VD and from 57 to 60 nm for LPT-VD. In addition to the good homogeneity of the samples, the ZP showed a comparable trend as it remained around −20 mV in the case of LP-VD and around −10 mV for LPT-VD (Table 5).

As is well known, during the storage, the liposomes are susceptible to aggregation due to their nanosized nature and high superficial area, producing heterogeneous particle size distribution. Both formulations remained stable as proved by their physical parameters.

As for freeze-dried nanoliposomes, immediately after preparation, the lyophilized products were rehydrated and analyzed by DLS. The physical parameters remained unchanged compared to those of the freshly prepared colloidal dispersions, with size of size 95.08 ± 1.02 nm and 0.28 ± 0.01 PdI for LP-VD, and 60.61 ± 0.73 nm and PdI 0.25 ± 0.01 for LPT-VD. After 20 days of storage at 25 °C and subsequent rehydration, the physical parameters resulted 97.65 ± 1.89 nm and 0.24 ± 0.01 PdI for LP-VD and 59.26 ± 1.58 nm and 0.25 ± 0.01 PdI for LPT-VD, providing that the process did not damage the liposomes, nor did it cause leakage, aggregation, or size changes.

#### 3.3.3. Gastrointestinal Stability

**Figure 2:**
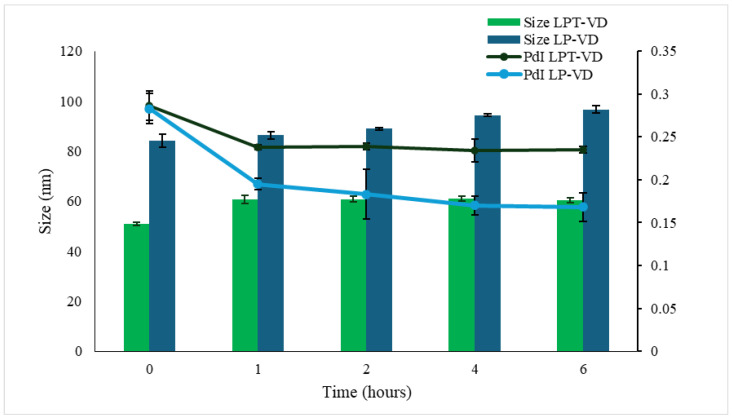
LPT-VD and LP-VD physical stability in SGF (2 h) and SIF (4 h). Data are reported as mean ± SD of n = 3.

Furthermore, physical stability was evaluated in SGF and SIF. Liposomes were first incubated in SGF for 2 h, followed by incubation in SIF for the next 4 h. Both formulations exhibited good stability in both conditions (Figure 2) with the size increasing from 85.8 nm to 105.7 nm for LP-VD, and from 50.86 nm to 60.37 nm LPT-VD; good homogeneity was retained. These findings indicate that, despite exposure to varying pH levels and ionic strength, both formulations had excellent physicochemical stability, maintaining size below 200 nm and PdI values below 0.3. The minimal size and PdI variations suggest that no significant aggregation or vesicle disruption occurred.

### 3.4. Dissolution Test

The study was conducted on three samples formulated in capsules: VD, LP-VD and LPT-VD freeze-dried products. 20 mg of VD was encapsulated in type 0 hard gelatin capsules. A quantity of LP-VD and LPT-VD corresponding to 20 mg of VD, were filled into type 00 capsules, due to their higher bulk volume.

**Figure 3:**
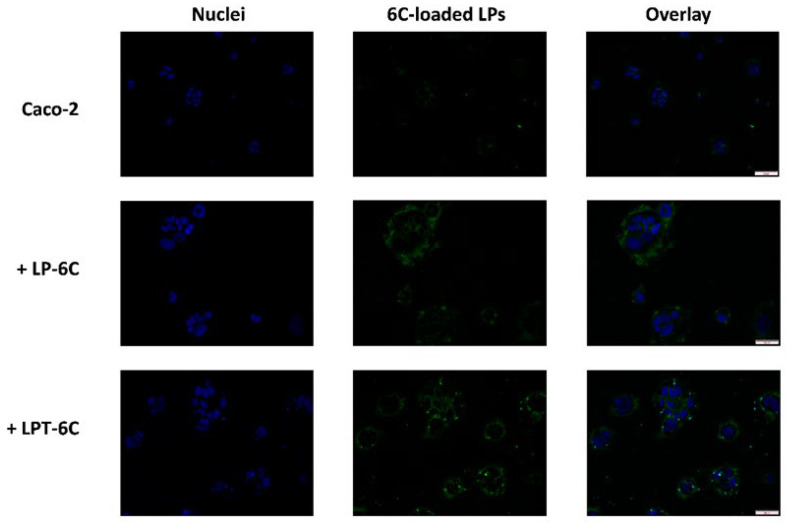
Cellular uptake of LP-6C and LPT-6C by Caco-2 cells after 2 h incubation at 37 °C. Images of nuclei stained with DAPI (blue), 6-Coumarin (green) and their overlay. Scale bar: 50 µm.

The dissolution profile of VD has a rapid initial release, with 17.25% of the molecule dissolved within the first hour and 26.40% after 2 h and it remains unchanged over the subsequent 24 h (Figure 3). Under these conditions, only approximately 4 mg of VD contained in the capsule were solubilized in the 900 mL of dissolution medium. A stability study of VD in the dissolution medium was conducted to verify whether the low dissolved fraction was due to concurrent degradation of VD. A solution (2 mg/mL) of VD in the dissolution medium containing 1% *v*/*v* of methanol was prepared. The solution was maintained at 37 °C under magnetic stirring. The initial concentration of VD in the solution was determined by HPLC analysis and resulted 0.02 mg/mL. Subsequent measurements were performed at various time points to monitor the stability of VD in the medium. After 6 h, approximately 50% of the VD was degraded, and complete degradation was observed after 48 h. Thus, the VD degradation plays a significant role in limiting its dissolution in the medium [31,32]. This finding further supports the validity of the data obtained with liposomal formulations.

**Figure 4:**
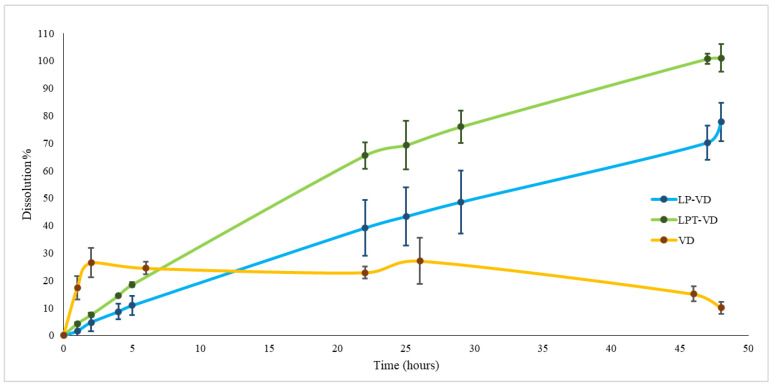
Dissolution release profiles of VD from a VD solution, freeze-dried LP-VD and LPT-VD in water (0.3% w/v SDS) at 37 °C. Data are reported as mean ± SD of n = 3.

Initially, the dissolution of VD from liposomes occurred more slowly than that of VD in solution. Specifically, after two hours, only 7.46% of the LPT-VD formulation had dissolved and 4.73% of the LP-VD formulation. The time required for rehydration of the lyophilized samples may explain the initially slower dissolution of VD. The amount of solubilized compound increased steadily, reaching complete dissolution after 48 h for LPT-VD and 77.68% for LP-VD (Figure 4).

Nanoliposomes positively influenced the dissolution of VD, as a consequence of improved solubility and stability.

### 3.5. Cellular Uptake Studies

Uptake studies were performed to highlight whether nanoliposomes could improve the penetration of VD into cells. For this purpose, labeled liposomes (LP-6C and LPT-6C) were prepared using 6C as fluorescent probe and the cellular uptake behavior was observed under fluorescence microscope. The EE% was 79.95 ± 5.44% for LP-6C and 73.4 ± 6.86% for LPT-6C. Furthermore, before the experiments, the stability of the probe inside the liposomes was evaluated, for a correct interpretation of the results. Both labeled nanoliposomes are stable, exhibiting a 6C release after 2 h of only 4.2% and 3.08% for LP-6C and LPT-6C, respectively. Then the vesicles can transport the compound into cells, where it is subsequently released following liposomal disruption [20,29].

Caco-2 cells were exposed for 2 h to the LP-6C and LPT-6C loaded with 0.5 mg/mL of 6C and diluted 1:1000 into a medium, and to a saturated solution of fluorescent probe. Figure 3 shows the fluorescence images of Caco-2 cell treated with LP-6C and LPT-6C after 2 h of incubation. The images revealed that 6C and the two liposomes can penetrate in the cells. The data show that cells treated with LP-6C and LPT-6C have a higher fluorescence intensity than cells treated with 6C. Furthermore, the fluorescence obtained following treatment with TPGS liposomes was greater than that of liposomes without TPGS.

Caco-2 cells, used to simulate the intestinal barrier, express on their surface the P-gp efflux pumps, as they are of tumor origin [34]. The increase in 6C uptake in cells treated with LPT-6C can be attributed to the inhibitory action of TPGS on these transporters. As illustrated in paragraph 3.1, TPGS is a polymer known for its ability to inhibit P-gp proteins [35]. This result suggests that liposomes formulated with TPGS could promote a greater intestinal absorption of the active ingredient in vivo compared to those without this component.

### 3.6. MTT and LDH Assays

**Figure 5:**
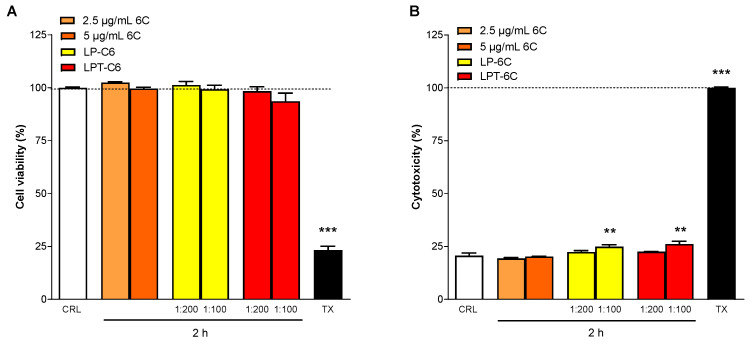
Caco-2 cell viability evaluated by MTT assay (**A**) and cytotoxicity by LDH assay (**B**) when exposed for 2 h to 6C (2.5 and 5 µg/mL) or LP-6C and LPT-6C (2.5 and 5 µg/mL). Data are expressed as percentage of control (CRL, medium) and triton-X (TX) which represent, respectively, the maximum cell viability and cell cytotoxicity. Values represent the mean ± SEM of at least three experiments performed in triplicate. ** p < 0.01 and *** p < 0.001 vs. CRL (one-way ANOVA followed by Dunnett’s test).

MTT and LDH assays were performed in the Caco-2 cell line to evaluate the effect of 6C and LP-6C and LPT-6C in cell viability and cytotoxicity. Caco-2 cells were incubated for 2 h with 6C, LP-6C, and LPT-6C at concentrations of 5 and 2.5 μg/mL, corresponding to 1:100 and 1:200 dilutions, respectively (Figure 5). The cells were then analyzed using various experimental approaches, including MTT and LDH release assays. In the MTT-based cell viability assays, no significant differences were observed between cells treated with 6-coumarin, LP-C6 or LPT-C6 at either dilution, compared to the vehicle-treated cells (Figure 5A). Caco-2 cells treated for 2 h with 6C showed no significant differences in LDH levels, an indicator of cell death and cytotoxicity, compared to the vehicle (Figure 5B). In contrast, LP-6C and LPT-6C exhibited significant cytotoxicity only at the 1:100 dilution (5 μg/mL), relative to the vehicle (Figure 5B).

**Figure 6:**
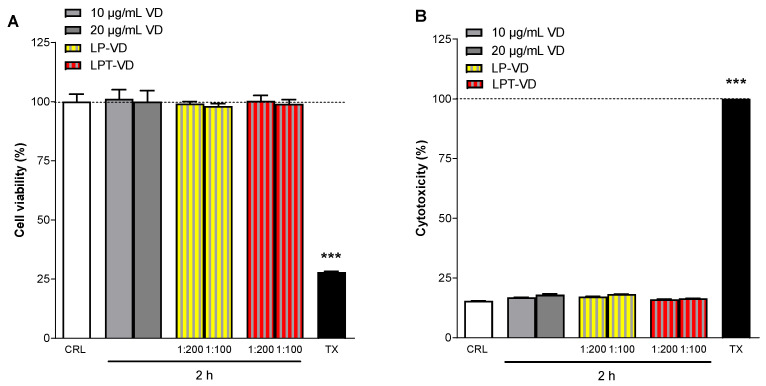
Caco-2 cell viability evaluated by MTT assay (**A**) and cytotoxicity by LDH assay (**B**) when exposed for 2 h to VD (10 and 20 µg/mL) or LP-VD and LPT-VD (10 and 20 µg/mL). Data are expressed as percentage of control (CRL, medium) and triton-X (TX) which represent, respectively, the maximum cell viability and cell cytotoxicity. Values represent the mean ± SEM of at least three experiments performed in triplicate. *** p < 0.001 vs. CRL (one-way ANOVA followed by Dunnett’s test).

Cell viability (MTT assay) and cytotoxicity (LDH assay) studies on Caco-2 cells were also performed using VD, LP-VD, and LPT-VD, following 2-h incubation at concentrations of 0.02 mg/mL and 0.01 mg/mL, corresponding to 1:100 and 1:200 dilutions, respectively. In cell viability assays performed using the MTT method, no significant differences were observed between cells treated with VD, or nanoliposomes at either dilution, relative to vehicle-treated cells (Figure 6A). Caco-2 cells treated for 2 h with VD showed no significant differences in LDH levels-an indicator of cell death and cytotoxicity- compared to the vehicle (Figure 6B). Similarly, LP-VD and LPT-VD, at both dilutions, did not show cytotoxic effects compared to the vehicle (Figure 6B). The data show that the VD liposomes appear to be less toxic, despite higher concentrations of VD free. In fact, neither LP-VD nor LPT-VD showed any cytotoxicity towards Caco-2 cells at any tested concentration.

## 4. Conclusions

Encapsulation of VD in nanoliposomes significantly improved the solubility, stability, and cellular uptake of VD, suggesting the potential use of these DDSs for the delivery of this vitamin. Nanoliposomes were produced successfully by thin film hydration-sonication method, with high encapsulation efficiency. The two formulations differ in the composition of the bilayer: both contain cholesterol and phosphatidylcholine as lipid components, but one also includes TPGS. The encapsulation in nanoliposomes of liposoluble vitamin reduced its degradation and provided stable aqueous dispersions. The solubility increased to 2 mg/mL. The greatest protective effect was shown at 4 °C and in the TPGS containing nanoliposomes. The freeze-drying improved stability even at 25 °C. The recovery remained at around 100% for 28 days at both temperatures. Stabilization was also evident in gastrointestinal environment; both LP-VD and LPT-VD protected VD in the aqueous dissolution medium, realizing a prolonged release. The results confirmed the safety of all formulations and an improved Caco-2 cellular uptake of VD, particularly in the case of LPT-VD, demonstrating the effect of TPGS as bioenhancer and inhibitor against the P-gp efflux pump.

## Figures and Tables

**Table 1:** Composition of nanoliposomes (weight ratio). PC: Phosphatidylcoline, CH: Cholesterol, TPGS: D-α-Tocopherol polyethylene glycol 1000 succinate, VD: vitamin D.

Formulation	PC	CH	TPGS	VD
LP	3	1	-	-
LPT	3	1	3	-
LP-VD	3	1	-	0.2
LPT-VD	3	1	3	0.2

**Table 2:** Physical characterization of LPT obtained with different PC:CH:TPGS ratio. Data are reported as mean ± SD of n = 3 different batches.

PC:CH:TPGS Ratio	Size (nm) ± SD	PdI ± SD
3:1:1	83.96 ± 0.40	0.22 ± 0.02
3:1:3	59.36 ± 0.70	0.26 ± 0.02
3:3:1	135.20 ± 0.26	0.22 ± 0.01

**Table 3:** Physical characterization of empty liposomes (LP, LPT) and VD-loaded liposomes (LP-VD, LPT-VD). Data are reported as mean ± SD of n = 5 different batches.

	Size (nm) ± SD	PdI ± SD	ZP ± SD (mV)	EE%
LP	69.98 ± 1.90	0.23 ± 0.01	−21.12 ± 3.24	-
LPT	59.36 ± 0.70	0.26 ± 0.01	−9.50 ± 3.57	-
LP-VD	85.50 ± 5.70	0.24 ± 0.06	−20.90 ± 4.37	95.76 ± 1.26%
LPT-VD	61.70 ± 3.90	0.26 ± 0.02	−9.45 ± 2.99	97.54 ± 3.24%

**Table 4:** Chemical stability of VD, LP-VD, LPT-VD, as colloidal dispersions, expressed as R%, stored under dark conditions at +4 °C. Data are reported as mean ± SD of n = 3.

Sample	1 Day	3 Days	4 Days	7 Days	14 Days	21 Days	28 Days
VD solution	86.46 ± 4.23	40.17 ± 3.4	39.28 ± 3.38	-	-	-	-
LP-VD	99.54 ± 2.42	99.85 ± 0.69	99.41 ± 3.13	98.95 ± 2.47	98.20 ± 2.79	96.53 ± 0.95	94.12 ± 5.25
LPT-VD	99.90 ± 2.75	99.65 ± 2.65	99.87 ± 4.49	99.35 ± 1.23	97.86 ± 1.22	96.89 ± 4.84	96.69 ± 4.94

**Table 5:** LP-VD and LPT-VD physical stability, as colloidal dispersion stored at +4° C. Data are reported as mean ± SD of n = 3.

		LP-VD			LPT-VD	
Time (Days)	Size	PdI	ZP	Size	PdI	ZP
0	84.72 ± 5.70	0.26 ± 0.01	−20.22 ± 0.93	57.20 ± 0.51	0.26 ± 0.00	−9.6 ± 0.6
1	84.31 ± 3.11	0.25 ± 0.01	−20.54 ± 0.35	57.97 ± 0.53	0.25 ± 0.01	−9.9 ± 0.3
5	85.13 ± 4.19	0.25 ± 0.01	−20.38 ± 0.26	57.99 ± 0.23	0.26 ± 0.00	−10.0 ± 0.2
7	88.52 ± 6.11	0.25 ± 0.02	−21.43 ± 1.10	58.89 ± 0.99	0.25 ± 0.01	−9.92 ± 0.3
14	87.23 ± 2.60	0.22 ± 0.25	−22.24 ± 1.18	57.83 ± 0.50	0.27 ± 0.00	−9.74 ± 0.3
21	93.14 ± 2.97	0.25 ± 0.00	−23.80 ± 0.70	58.70 ± 0.61	0.27 ± 0.01	−12.2 ± 1.4
28	98.31 ± 3.23	0.25 ± 0.02	−26.42 ± 0.69	60.12 ± 0.72	0.28 ± 0.01	−12.9 ± 1.5

## Data Availability

The data presented in this study are available on request from the corresponding author.

## Supplementary Materials

The following supporting information can be downloaded at: https://www.mdpi.com/article/10.3390/pharmaceutics17101244/s1, Figure S1. HPLC profile of VD at 265 nm; Figure S2. LP-VD size distribution plot; Figure S3. LP-VD Zeta Potential distribution plot; Figure S4. LPT-VD size distribution plot; Figure S5. LPT-VD Zeta Potential distribution plot.
