# Supplementary material for: Vitamin D Nanoliposomes to Improve Solubility, Stability, and Uptake Across Intestinal Barrier
Source: Pharmaceutics. 2025 Sep 23;17(10):1244. doi: 10.3390/pharmaceutics17101244 (PMC12566816; doi:10.3390/pharmaceutics17101244)
Supplement: Supplementary file 1 [file pharmaceutics-17-01244-s001.zip › pharmaceutics-3809591-supplementary.pdf]

Supplementary material

Vitamin D Nanoliposomes to Improve Solubility, Stability, and Uptake Across Intestinal Barrier

Cosimo Landi [1], Elisa Landucci [2], Costanza Mazzantini [2], Rebecca Castellacci [1] and Maria Camilla Bergonzi [1,*]

[1] Department of Chemistry, University of Florence, Via Ugo Schiff 6, Sesto Fiorentino, 50019 Florence, Italy; cosimo.landi@unifi.it (C.L.); rebecca.castellacci@unifi.it (R.C.)

[2] Department of Health Sciences, University of Florence, Viale Pieraccini 6, 50139 Firenze, Italy; elisa.landucci@unifi.it (E.L.); costanza.mazzantini@unifi.it (C.M.)

* Correspondence: mc.bergonzi@unifi.it; Tel.: +39 055-4573678

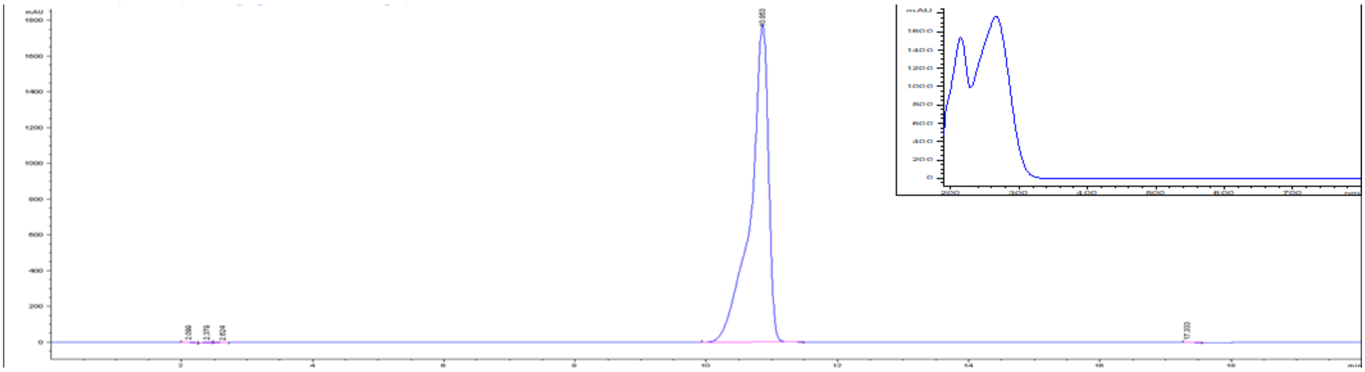

Figure S1. HPLC profile of VD at 265 nm.

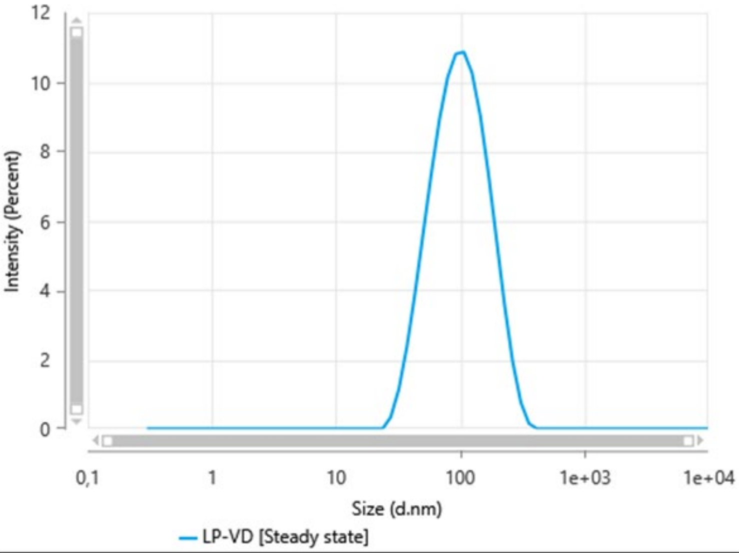

[redacted]

Name	Mean	Standard Deviation	RSD	Minimum	Maximum
Z-Average (nm)	89,43	-	-	89,43	89,43
Polydispersity Index (PI)	0,2149	-	-	0,2149	0,2149
Peak 1 Mean by Intensity ordered by area (nm)	111,9	-	-	111,9	111,9
Peak 1 Area by Intensity ordered by area (%)	100	-	-	100	100

Figure S2. LP-VD size distribution plot

			Mean (mV)	Area (%)	St Dev (mV)
Zeta Potential (mV):	-20,6	Peak 1:	-20,6	100,0	7,07
Zeta Deviation (mV):	7,07	Peak 2:	0,00	0,0	0,00
Conductivity (mS/cm):	0,0360	Peak 3:	0,00	0,0	0,00

Result quality : Good

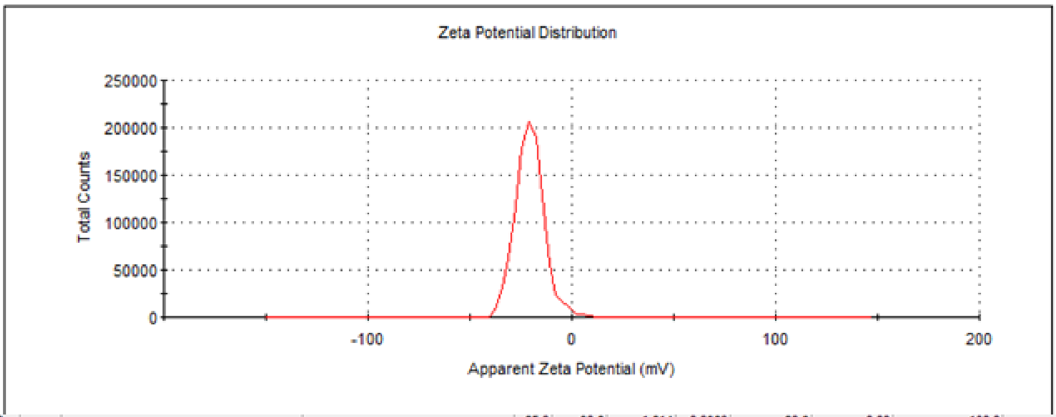

Mean 22				25,0	-20,6	-1,614	0,0360	-20,6	0,00	100,0	0,0
Std Dev											
RSD %											
Minimum				25,0	-20,6	-1,614	0,0360	-20,6	0,00	100,0	0,0
Maximum				25,0	-20,6	-1,614	0,0360	-20,6	0,00	100,0	0,0

Figure S3. LP-VD Zeta Potential distribution plot.

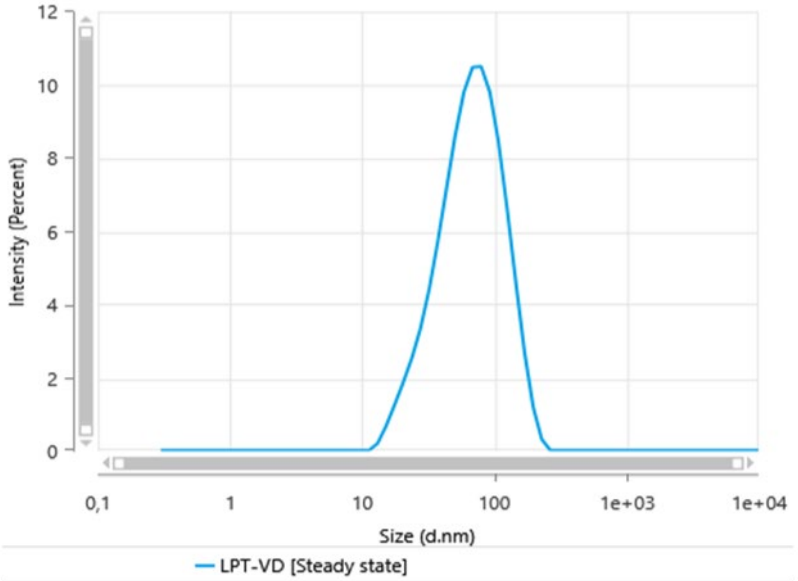

[redacted]

Name	Mean	Standard Deviation	RSD	Minimum	Maximum
Z-Average (nm)	57,17	-	-	57,17	57,17
Polydispersity Index (PI)	0,2252	-	-	0,2252	0,2252
Peak 1 Mean by Intensity ordered by area (nm)	75,45	-	-	75,45	75,45
Peak 1 Area by Intensity ordered by area (%)	100	-	-	100	100

Figure S4. LPT-VD size distribution plot.

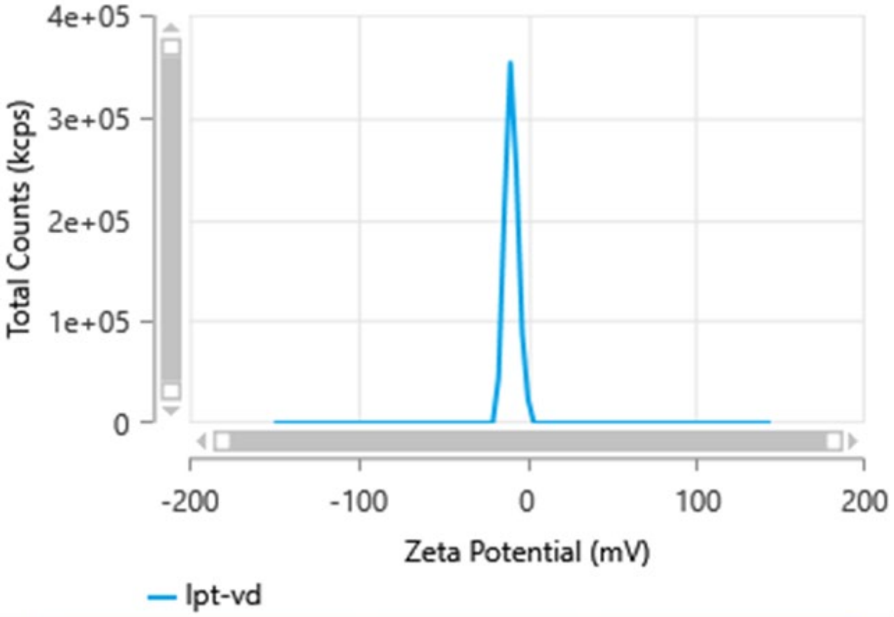

Name	Mean	Standard Deviation	RSD	Minimum	Maximum
Zeta Potential (mV)	-8,962	-	-	-8,962	-8,962
Conductivity (mS/cm)	0,02168	-	-	0,02168	0,02168
Wall Zeta Potential (mV)	-3,28	-	-	-3,28	-3,28
Quality Factor	1,355	-	-	1,355	1,355
Zeta Peak 1 Mean (mV)	-8,962	-	-	-8,962	-8,962

Figure S5. LPT-VD Zeta Potential distribution plot.
